# Supplementary material for: Shark mandible evolution reveals patterns of trophic and habitat-mediated diversification
Source: Commun Biol. 2023 May 8;6:496. doi: 10.1038/s42003-023-04882-3 (PMC10167336; doi:10.1038/s42003-023-04882-3)
Supplement: Supplementary file 2 — Supplementary Information [file 42003_2023_4882_MOESM2_ESM.pdf]

Shark mandible evolution reveals patterns of trophic and habitat-mediated diversification

Supplementary Information

Faviel A. López-Romero[1,2*], Sebastian Stumpf[1], Pepijn Kamminga[3], Christine Böhmer[4,5], Alan Pradel[6], Martin D. Brazeau[7,8], Jürgen Kriwet[1]

[1] University of Vienna, Faculty of Earth Sciences, Geography and Astronomy, Department of Palaeontology, Evolutionary Morphology Research Group, Josef-Holaubek-Platz 2, 1190 Vienna, Austria.

[2] University of Vienna, Vienna Doctoral School of Ecology and Evolution (VDSEE), Djerassiplatz 1, 1030 Vienna, Austria

[3] Naturalis Biodiversity Center, Darwinweg 2, 2333 CR, Leiden, The Netherlands

[4] MECADEV UMR 7179 CNRS/MNHN, Département Adaptations du Vivant, Muséum National d'Histoire Naturelle, CP 55, 57 rue Cuvier, 75231 Paris, France.

[5] Department für Geo- und Umweltwissenschaften und GeoBio-Center, Ludwig-Maximilians-Universität München, Richard-Wagner-Straße 10, 80333 München

[6] CR2P, Centre de Recherche en Paléontologie – Paris, Muséum National d'Histoire Naturelle – Sorbonne Université - CNRS, CP 38, 57 rue Cuvier, F75231 Paris Cedex 05, France.

[7] Department of Life Sciences, Imperial College London, Silwood Park Campus, Ascot, SL5 7PY, UK.

[8] Department of Earth Sciences, The Natural History Museum, Cromwell Road, London, SW7 5BD, UK

Supplementary Methods

Landmarking.

Six fixed landmarks were digitized on each specimen. In between the landmarks we placed sliding landmarks to capture the full extent of the curvature displayed in the jaws. Landmark 1 correspond to the upper medial symphysis, landmark 2 is the posterior end of the dental groove, landmark 3 is the medial quadratomandibular joint, landmark 4 is the posterior quadratomandibular joint, landmark 5 is the lower symphysis and landmark 6 is the inner lower symphysis. Between landmark 1 and 2 there are 8 sliding landmarks to capture the extent of the dental groove along the jaw. For the landmarks 2 and 3 there are 5 landmarks to describe the curve between the end of the dental groove and the quadratomandibular joint. Two curves encircle landmarks 3 and 4, in the medial view 7 sliding landmarks were used to describe the perimeter comprising the medial quadratomandibular joint and the lateral quadratomandibular joint, while on the ventral view 8 sliding landmarks were used to describe the mandibular knob. For landmarks 4 and 5, 15 sliding landmarks were used to capture the lower curvature of the jaw from the posterior quadratomandibular joint to the lower symphysis. Finally, from landmarks 2 and 6, 8 semilandmarks were used for the curve of the dental sulcus from the posterior end of the dentition to the lower ventral symphysis. This configuration was captured for every specimen. Additionally, in the specimen of *Carcharhinus acronotus*, this configuration was used to also place surface landmarks on the medial view of the jaw, with 43 surface landmarks this configuration was used as a template, for a total of 100 coordinates. Initially, a configuration with 257 coordinates was used, however this was re-estimated with the LASEC function (1), which indicated that around 100 coordinates were sufficient to describe the shape among the number of specimens (Supplementary figure S2). The complete configuration of landmarks and semilandmarks for all the specimens was then imported as a PTS file for subsequent analysis.

Initially with the reference template built in *Carcharhinus acronotus*, we used the function createAtlas in the R package Morpho (2). This function allowed us to have a reference for the rest of the coordinates configurations in the other specimens and designate specific coordinates to be treated as fixed, curve or surface. We then built an array object from the PTS file which was resampled to remove duplicated landmarks (especially in the connection extremes of the curves used), and this array was used with the reference atlas with the placePatch function. This function was used with varying parameters since the scale can make the arrangement of surface landmarks to displace them and thus not capturing the surface shape. The final parameters for the placePatch function designated were: ray = TRUE, inflate = 3.5, tol = 5, relaxpatch = FALSE. The array of coordinates finally was then used for the geometric morphometric analyses.

Diet and ecology data

We updated the data from the work of Cortes (3); we first checked the raw values. In some cases, on the original table the values can go up to 100.1 % which is unreliable. In some of the cases this can be caused by an automatic rounding of the values which have decimals as 0.66667 to be equal to 0.7. Therefore, we first corrected such instances to have adjusted values. We then removed the species for which we do not have a specimen with jaws

reconstruction. In some specific cases we replaced a species feeding data with the closest relative for which stomach content studies have been performed. This was only done for a few specimens which were underrepresented within an order. Such instances are highlighted in the supplementary data table 1 with their respective reference. With the prey categories described by Cortes (3) we complemented from the literature for other species and create a table with percentages used for the Index of Relative Importance (Supplementary Data Table 2). The final table consist of 75 species. With this table we performed a clustering with the Bray Curtis Index followed by clustering by UPGMA. However, after the first observation of the cluster dendrogram (Supplementary Figure S3) we notice certain species affect the outcome of the average silhouette width (with the find_k function in dendextend R package (4). For this reason, we ran the analysis again excluding these species for which a 100% of diet component is present (Supplementary figure S4). Overall, the defined groups were consistent in the second dendrogram, however, other groups like the Generalists and Crustacean consumers required further assessment. We decided to keep the Generalist group since it shows a wider prey spectrum compared to the Crustacean consumers as shown in the heatmap. This groups were used as categorical variables for other comparisons with the shape and size data (Supplementary Data Table 3). In the case of the trophic levels, we first obtained the information from the Index of Relative Importance of each of the used species as reported (http://www.fishbase.org/) (5), as in the case of stomach content information, some species do not have an exact estimation of the trophic level. In these particular cases the reported value is estimated from the value on close relatives of the species. This information is also highlighted in the supplementary Data Table 1. The trophic level was then used as a categorical variable as proposed by Froese (6), with values lower than 3.8 as Low-level predator (LP; n = 20), from 3.81-4.2 as Mesopredators (MP; n = 41), and values greater than 4.2 as top predators (TP; n = 29). But also, the raw values of trophic level were also used with the shape variables for comparison. The information on ecological lifestyle determinate from the ecomorphotypes (7), which were adapted in Dulvy et al (8). In this sense, the reef species are usually found with complex environments, either coral or rocky. The shelf species are found in the intertidal zone and up to 200m depth, but without a highly 3D complex environment. The pelagic species are found mostly in open sea, and less than 200m depth. And finally, the deep sea are found below 200m depth. In total we have reef associated (n = 28), shelf (n = 24), pelagic (n = 17), and deep-sea (n = 21) (Supplementary Data Table 3).

Biomechanical and shape comparison

We selected from the aligned coordinates those which could extract information related to the in-lever and out-lever, in order to estimate the mechanical advantage of jaw opening. The ratio was estimated by measuring the interlandmark distance between Landmark 1 and Landmark 6 divided by the interlandmark distance between Landmark 1 and Landmark 2. The second comparison was in relation to the torsional resistance. To estimate this measurement we used the interlandmark distance between Landmark 5 and 6 divided by interlandmark distance between Landmark 1 and 5, this value was then log transformed to reduce the difference with large specimens. The mechanical advantage estimated was then plotted against PC1, while the torsional resistance was plotted against PC2. This was done in order to corroborate the shape variation on Procrustes for each PC. We then estimated the correlation between these ratios and their corresponding PC. To measure the interlandmark

distance the interlmkdist function in geomorph (v. 4.0.4) (9), and the correlation with ggpubr (v. 0.4.0) (10).

Supplementary Figures

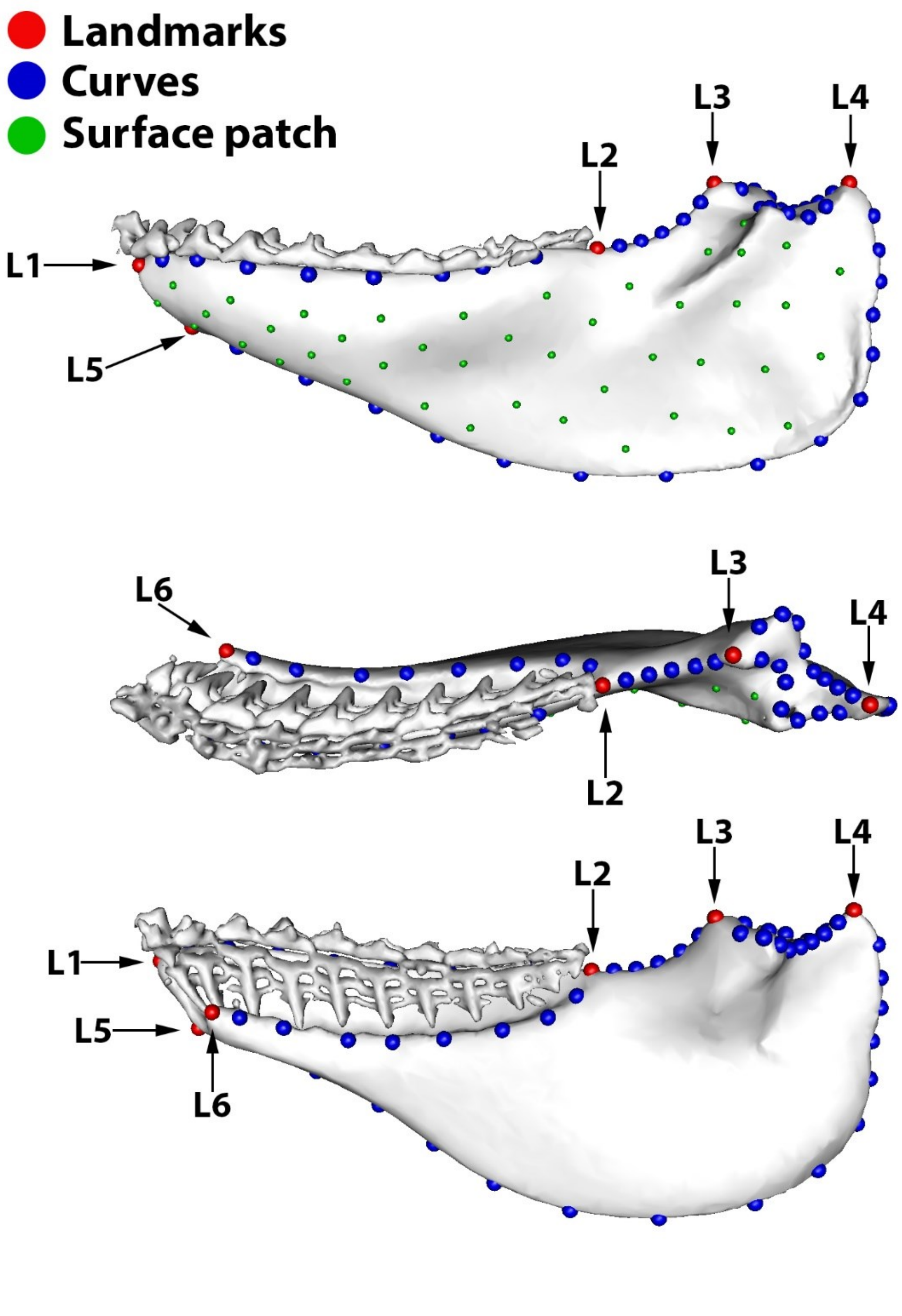

Supplementary Figure S1. Landmarks and sliding landmarks configuration on the lower jaw of *Carcharhinus acronotus*. Red dots represent true landmarks at the (L1) upper symphysis, (L2) posterior boundary of the dental groove, (L3) anterior quadratojugal joint, (L4) posterior quadratojugal joint, (L5) lower symphysis, and (L6) inner symphysis. The blue does denote curves along the defined landmarks. Green dots are surface landmarks over the lateral view of the lower jaw. No surface landmarks were used in the inner part of the jaw since some reconstructions were often incomplete in this area.

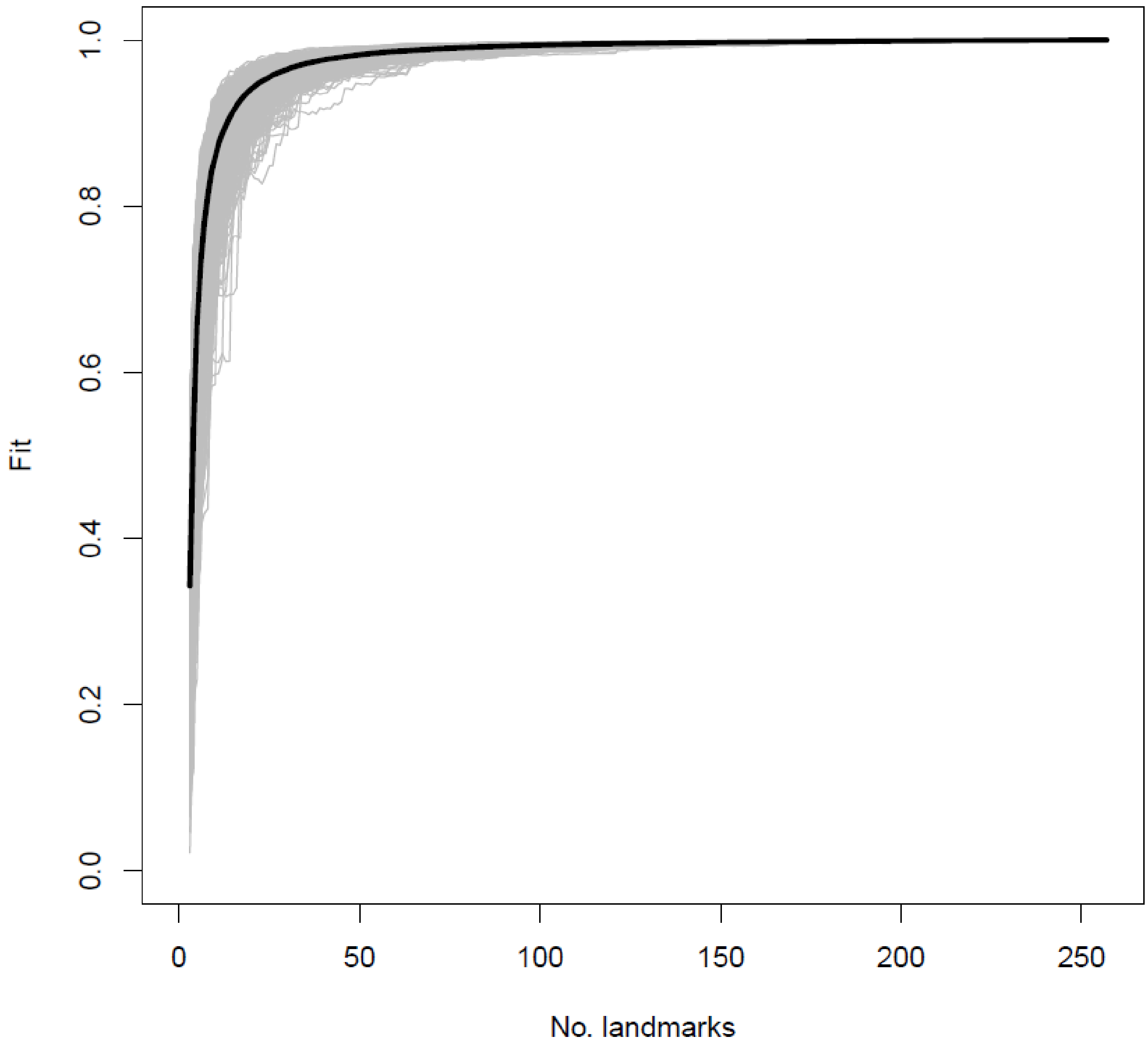

Supplementary Figure S2. Sampling curve from LaSEC on the 257 3D landmark coordinates. Grey lines indicate iterations fitted values. Solid line indicates the median fit value.

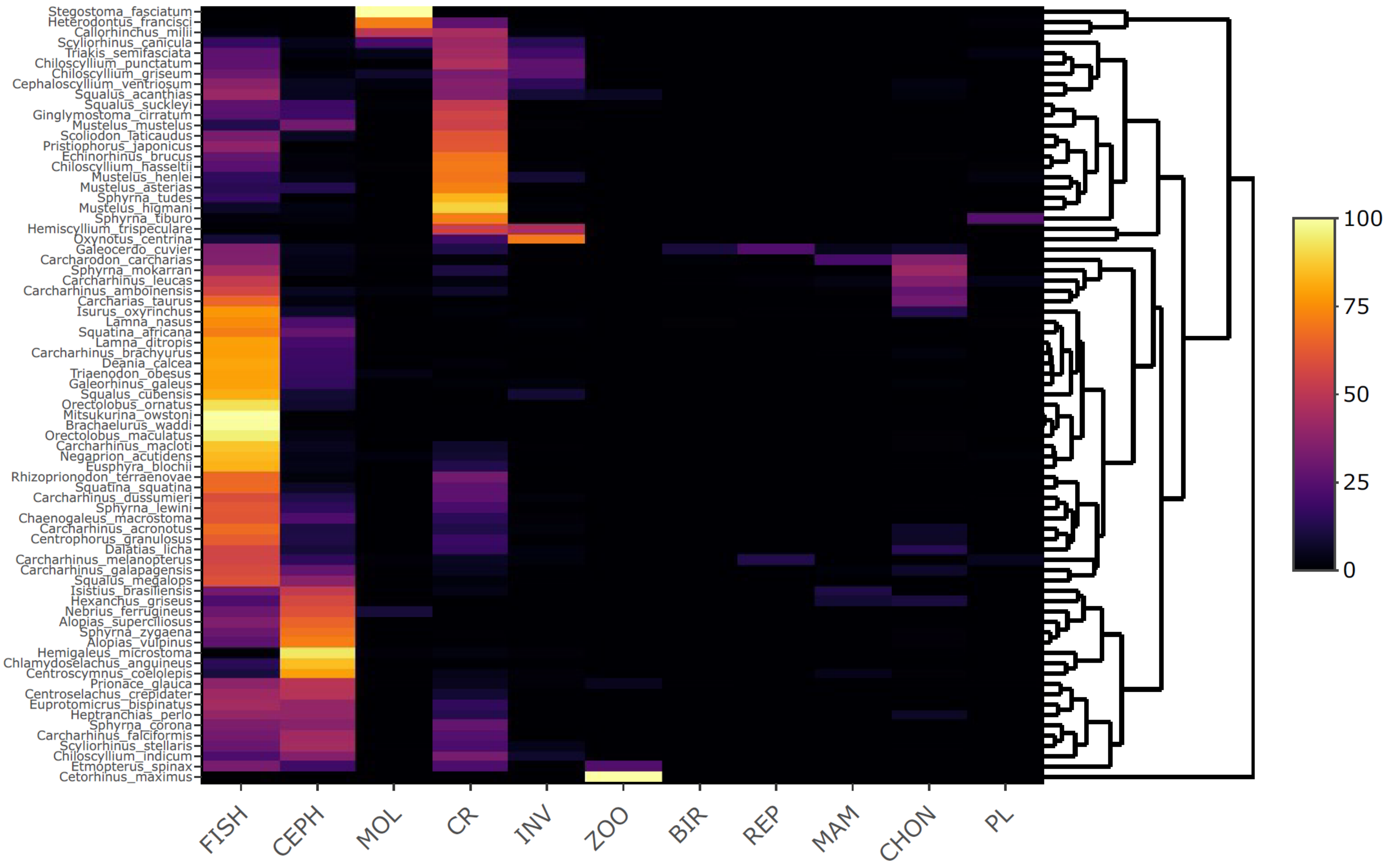

Supplementary Figure S3. Hierarchical cluster analysis of the dissimilarity of diet preferences among the sharks studied. Barplots on the right indicate the %IRI for each prey category reported for each species. Complete data with monospecific diets. Colour code indicates BIR = Bird; CEPH = Cephalopod; CHON = Chondrichthyan; CR = Crustacean; FISH = Bony fish; INV = Invertebrate; MAM = Mammal; MOLL = Hard shelled mollusc; PL = Plant; REP = Reptile and ZOO = Zooplancton.

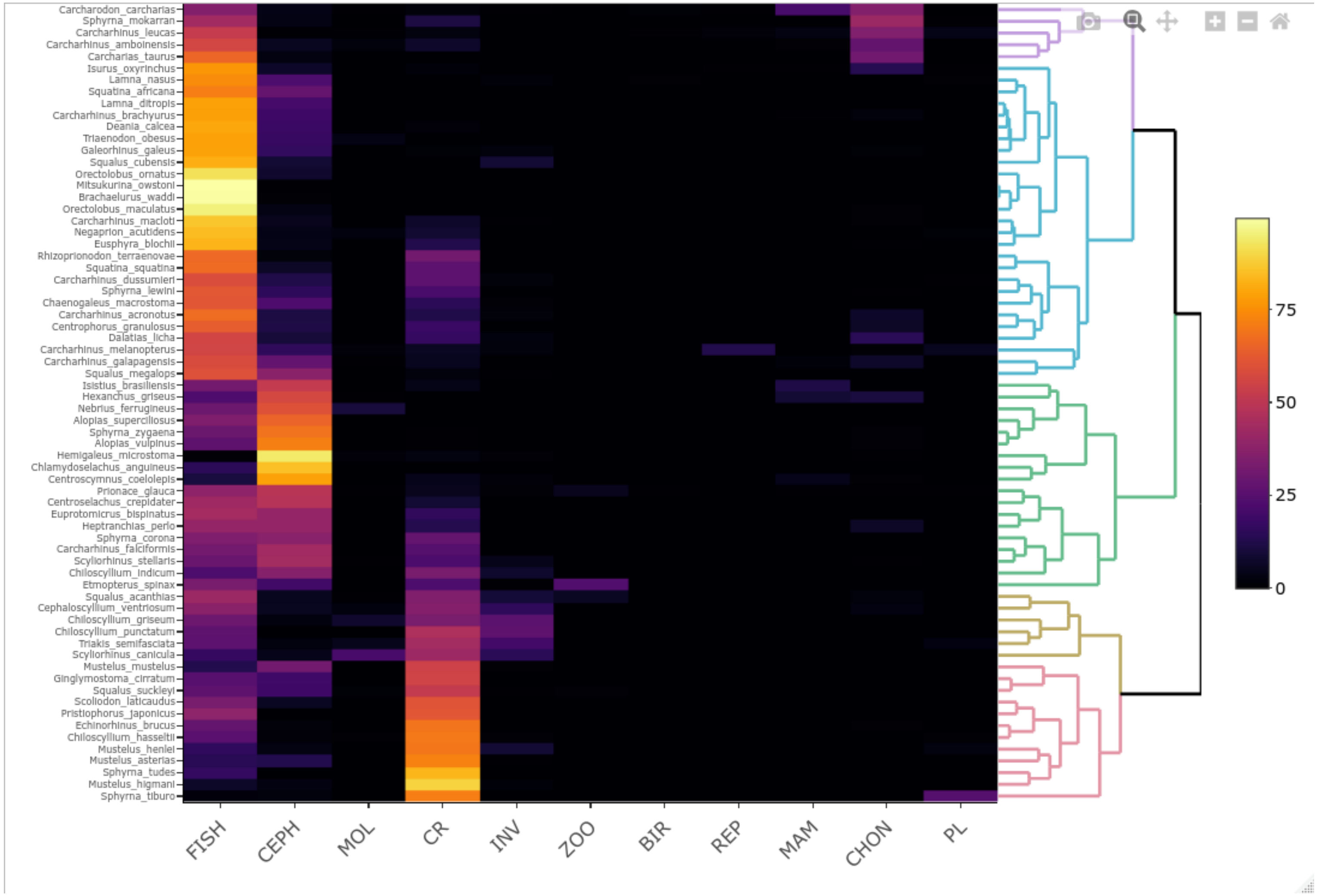

Supplementary Figure S4. Hierarchical cluster analysis of the dissimilarity of diet preferences for the species without mono-specific diet to observe arrangements in the clustering for larger groups.

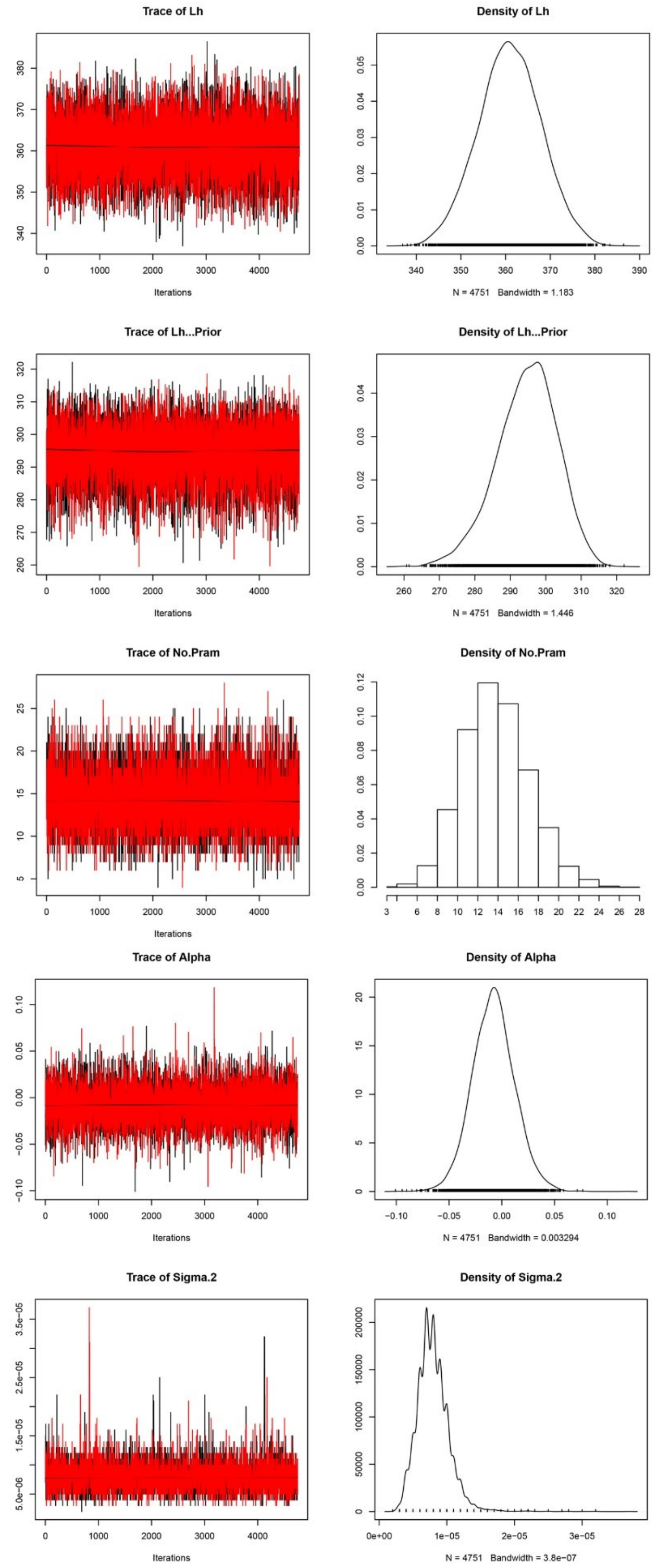

Supplementary Figure S5. Trace plots of two independent runs for the BayesTraits analysis, results from the Gelman-Rubin's convergence diagnostic and effective sample size in tables S1 and S2.

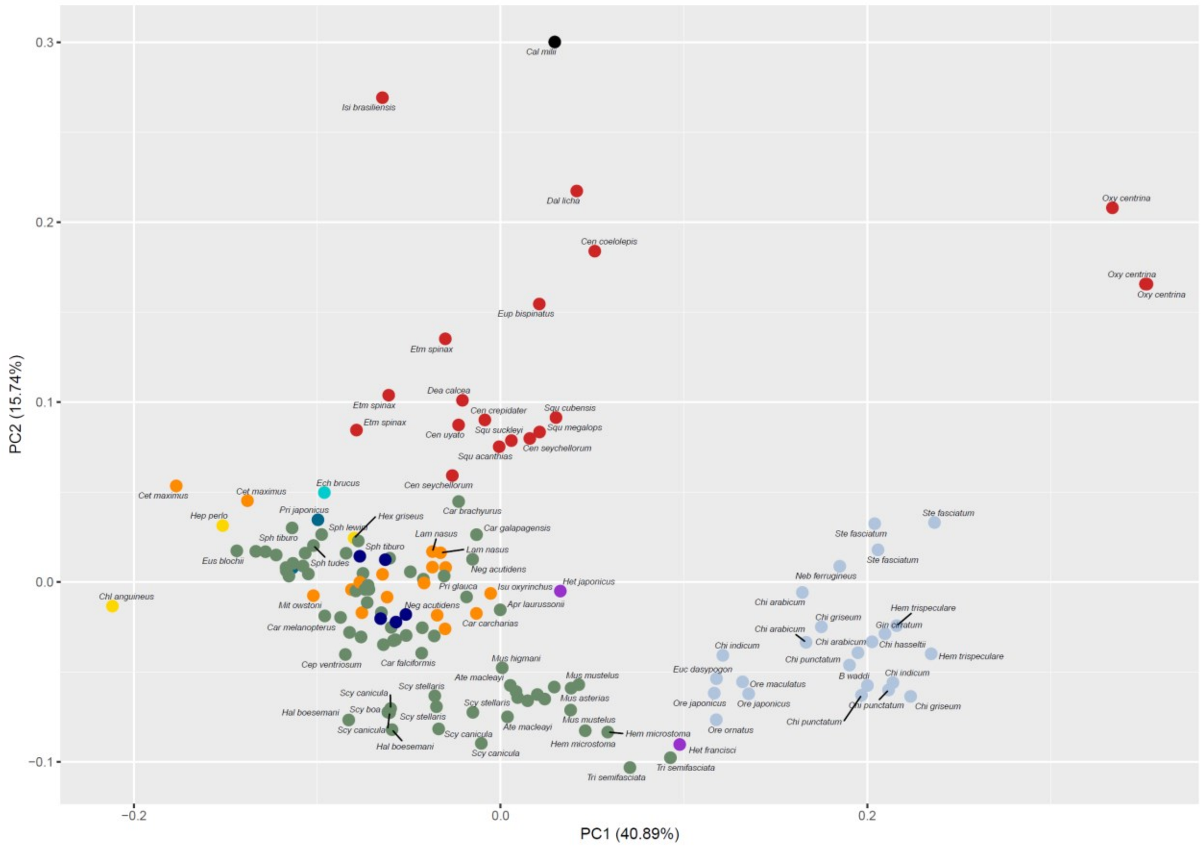

Supplementary Figure S6. Jaw shape variation along Principal Component 1 and 2. Coloured circles show the order as indicated in Figure 1 of main manuscript. Labels on each point indicate the species for each specimen.

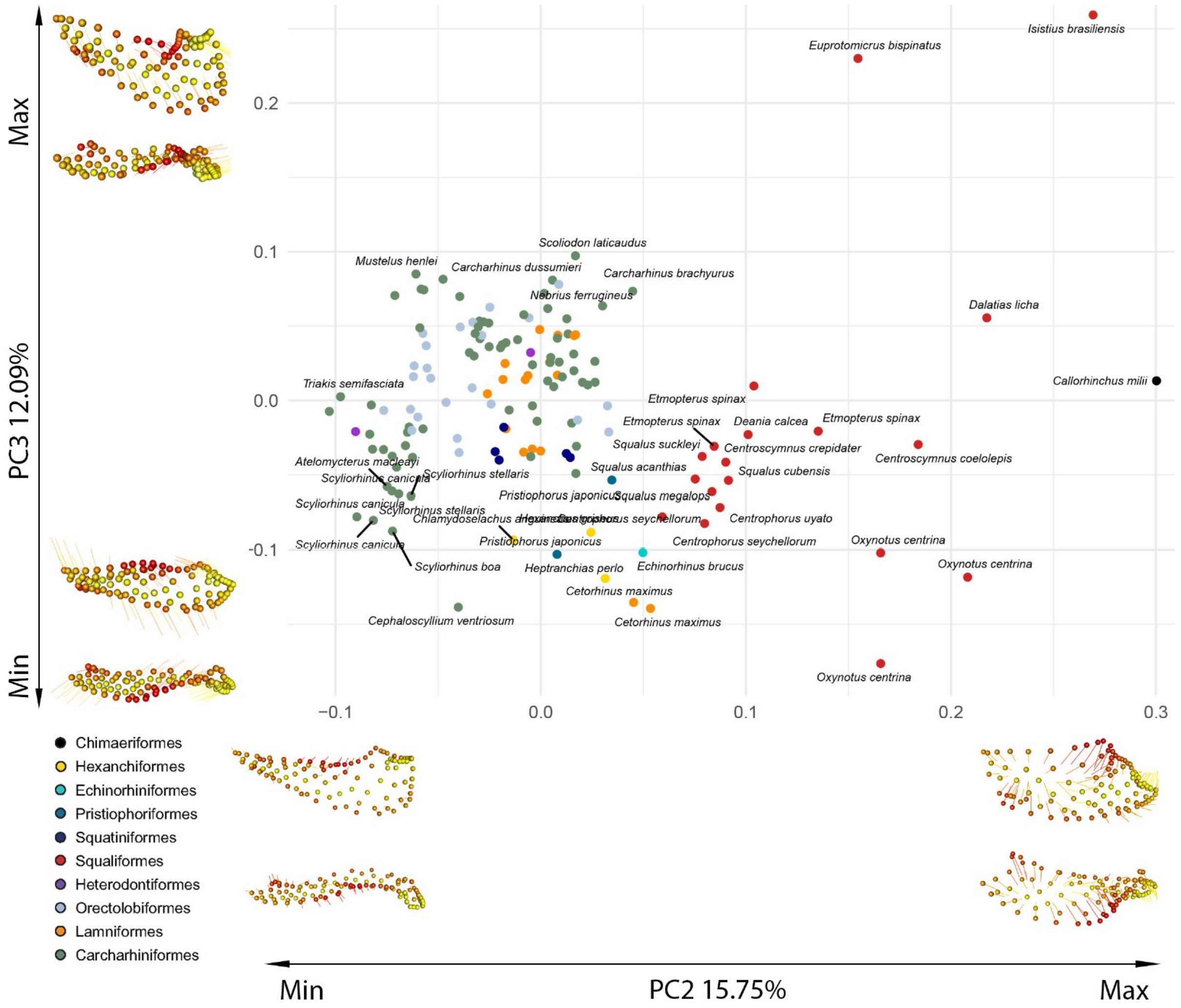

Supplementary Figure S7. Jaw shape variation along Principal Component 2 and 3. Coloured circles indicate the order.

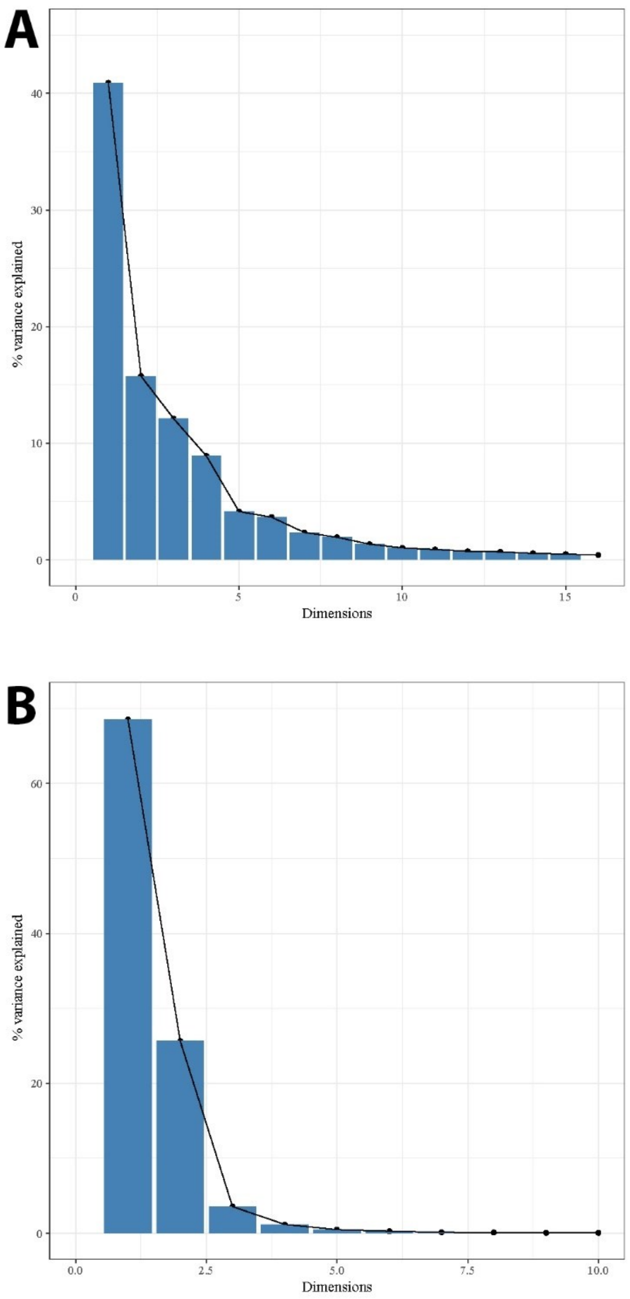

Supplementary Figure S8. Scree plots of the variance explained (95%) by the components for the whole set of specimens (n = 145) (A), and the phylogenetic aligned PCA (89 species) (B).

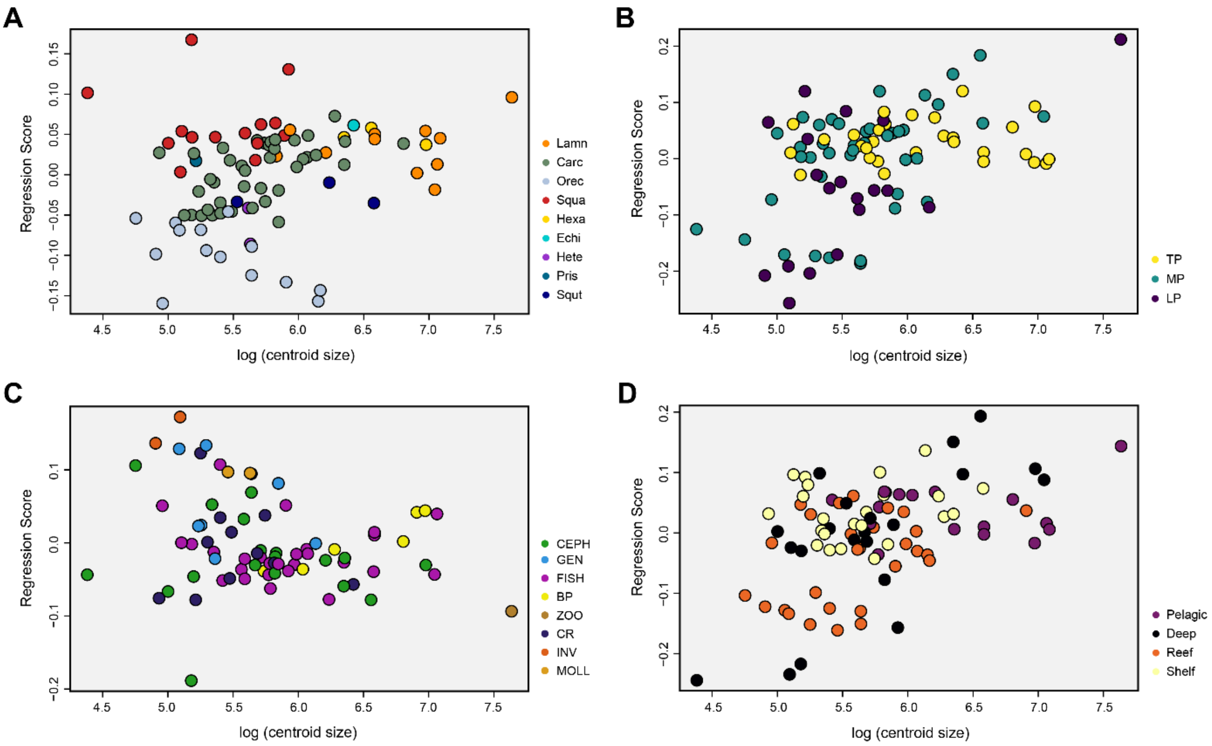

Supplementary Figure S9. Log(centroid size) against the multivariate regression of the Procrustes coordinates (regression score) of shape for each species in the four analysed groups. (A) Orders (Lamn = Lamniformes; Carc = Carcharhiniformes; Orec = Orectolobiformes; Squa = Squaliformes; Hexa = Hexanchiformes; Echi = Echinorhiniformes; Hete = Heterodontiformes; Pris = Pristiophoriformes; Squt = Squatiniformes), (B) trophic levels (TP = Top Predator; MP = Mesopredator; LP = Low-level Predator), (C) diet groups, and (D) habitat (CEPH = Cephalopods; GEN = Generalist; FISH = Bony fishes; BP = Big Predator; ZOO = Zooplankton; CR = Crustaceans; INV = Invertebrates; MOLL = Hard shelled molluscs).

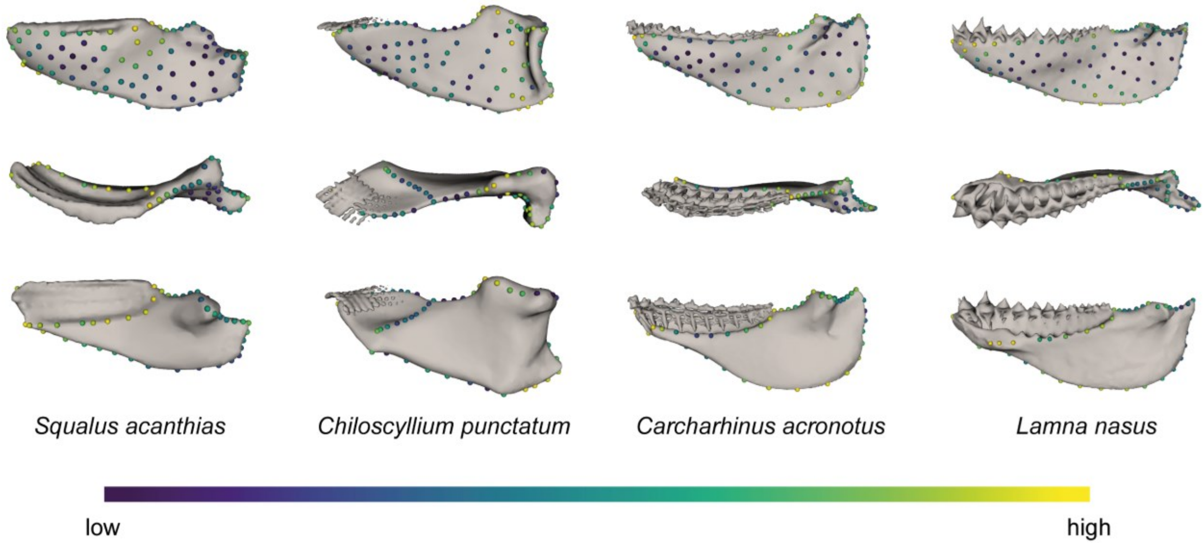

Supplementary Figure S10. Evolutionary rate per landmark for the main orders of sharks analysed. Landmarks and semilandmarks configurations on the jaws of representative orders in lateral (top row), dorsal (middle row) and buccal (bottom raw) view. Colder colours indicate low evolutionary rate and warmer colour indicate faster evolutionary rate.

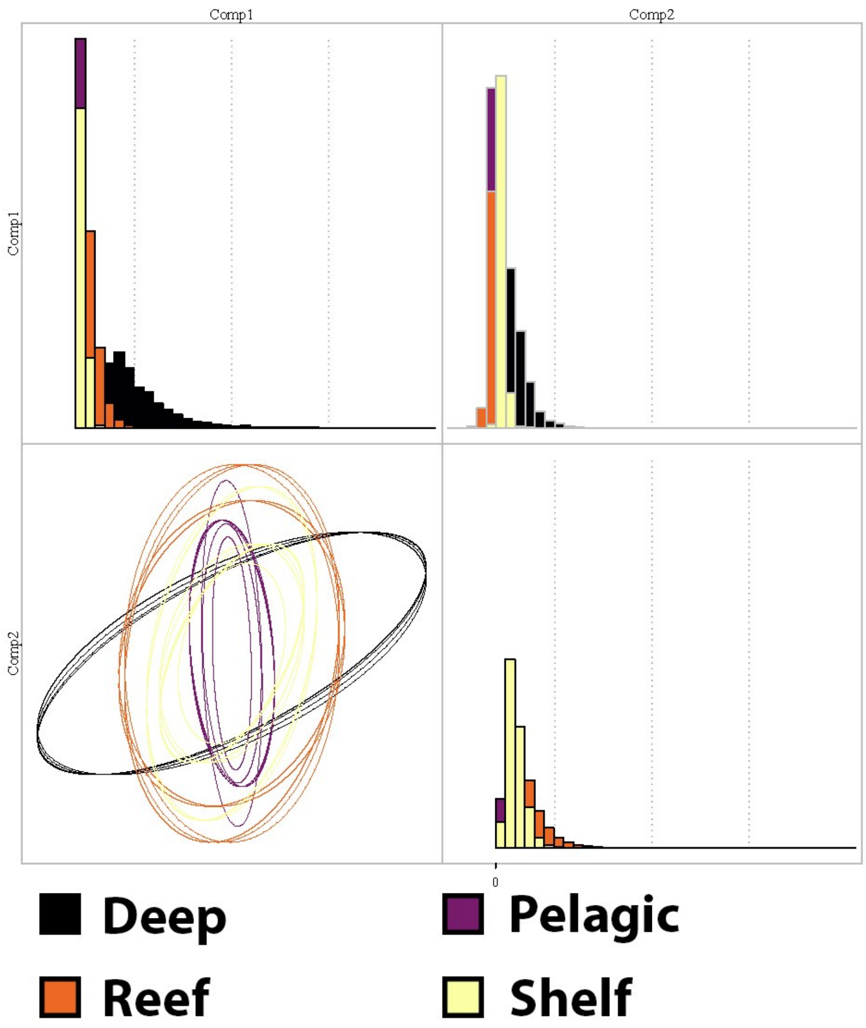

Supplementary Figure S11. Posterior distribution of parameter estimates using ratematrix. Upper left and lower right panels show the variances of each trait, Principal component 1 and2 respectively. Upper right panel shows pairwise covariation of traits. Lower left panel, samples of the posterior distribution within the 95% of credible interval of the distribution.

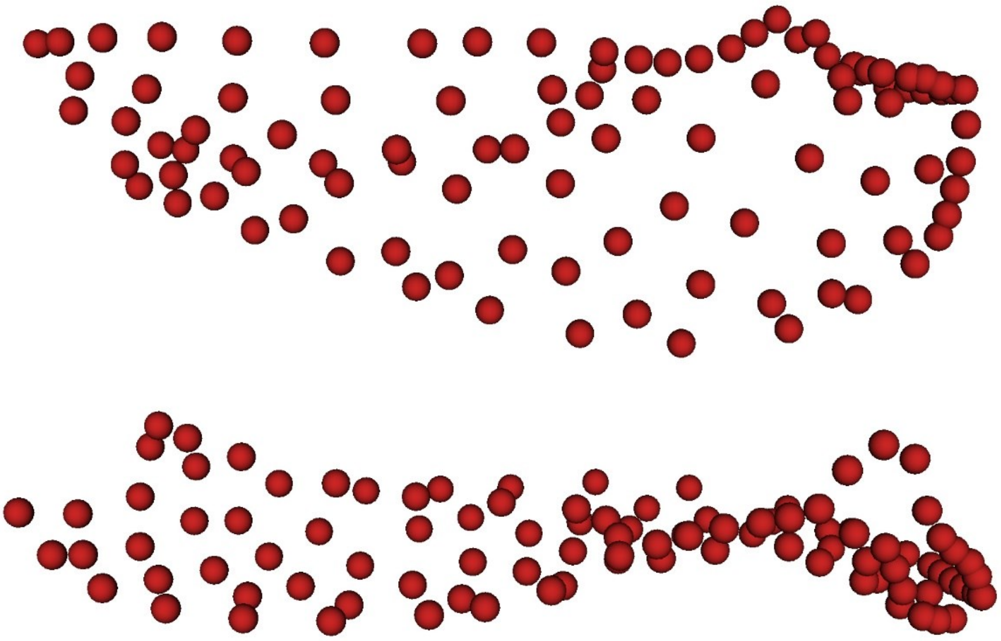

Supplementary Figure S12. Ancestral shape reconstruction with all the species, including *Callorhinchus milii*. Displayed in lateral (upper) and dorsal (lower) view.

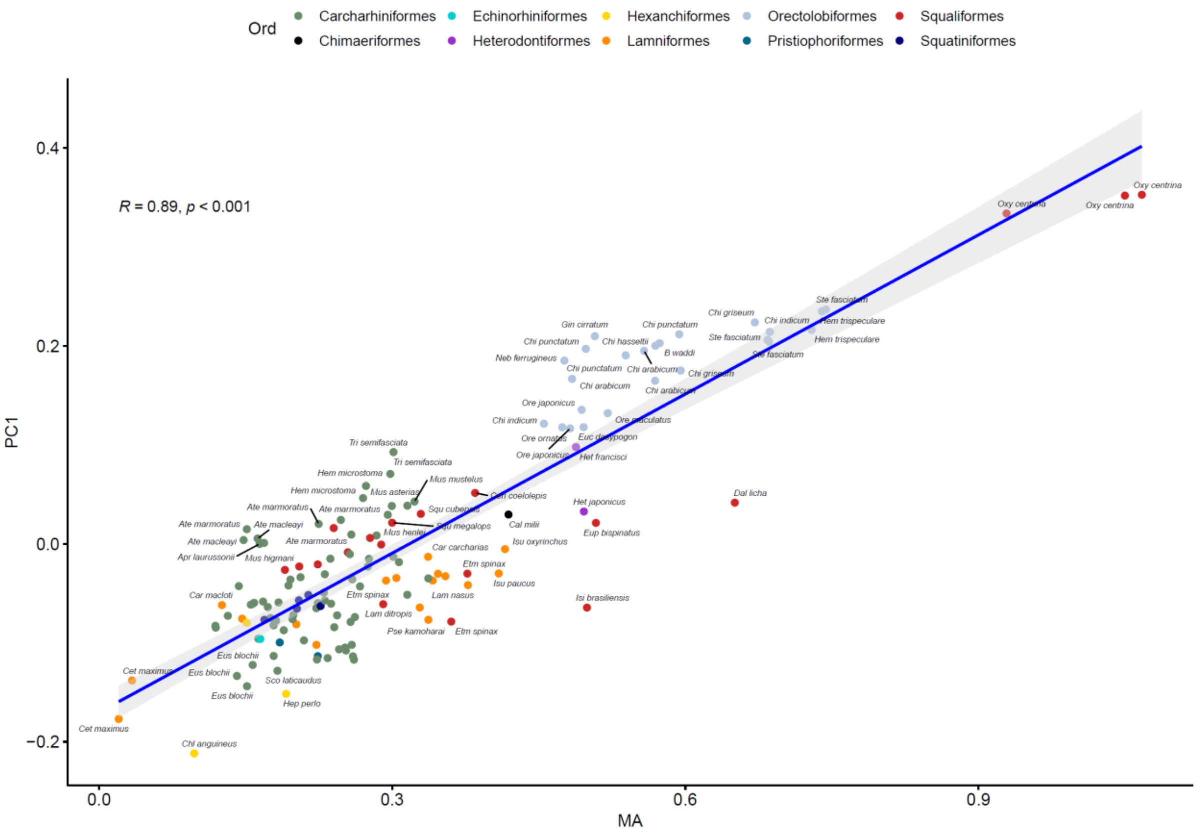

Supplementary Figure S13. Scatter plot of the mechanical advantage (MA) against PC1 scores in figure 1 of the main text. Pearson correlation estimated and P value indicated.

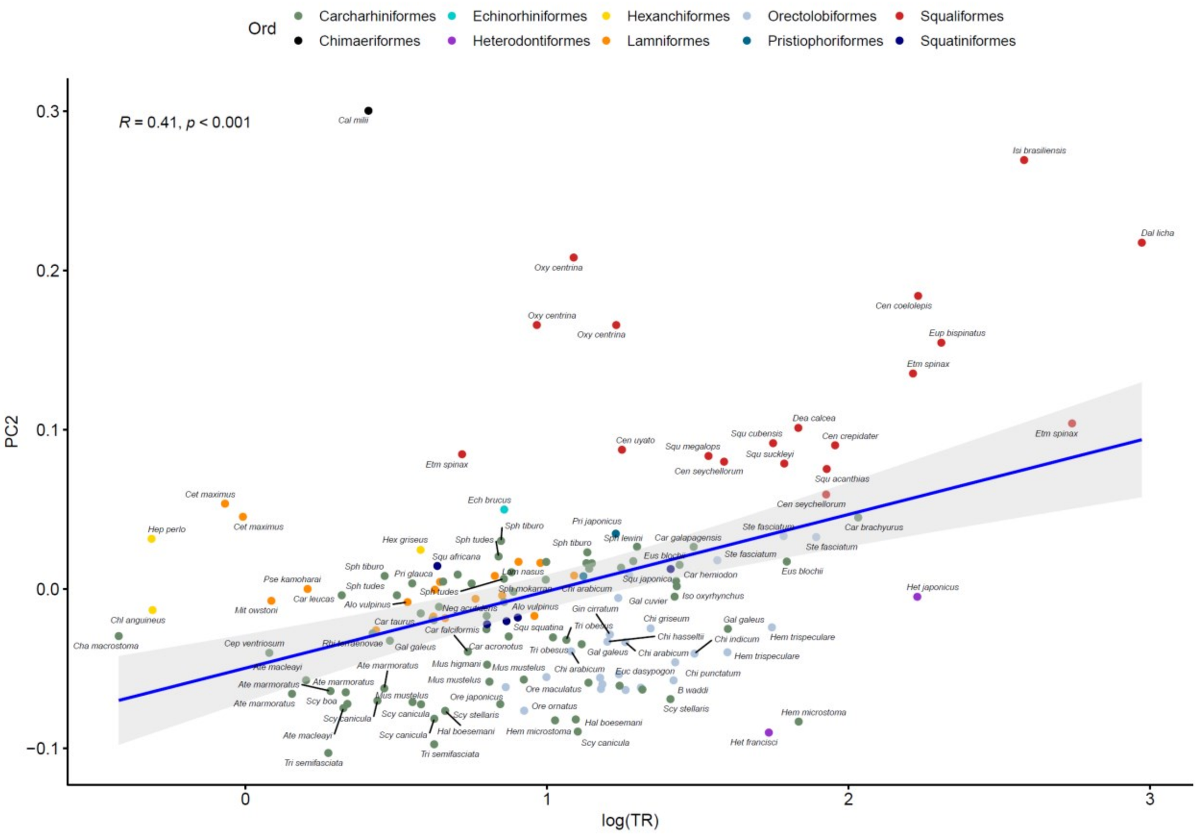

Supplementary Figure S14. Scatter plot of the logarithm of Torsional Resistance (log(TR)) against PC2 scores in figure 1 of the main text. Pearson correlation estimated and P value indicated.

Supplementary Tables

	AIC_ARD	logLik_ARD	AIC_SYM	logLik_SYM	AIC_ER	logLik_ER_Die
Diet	226.36306	-93.18153	**219.10526**	-99.55263	223.62904	-110.81452
Ord	100.40004	-20.20002	74.77314	-22.38657	**53.97350**	-25.98675
Hab	194.92703	-85.46352	**188.95130**	-88.47565	197.84734	-97.92367
TrL	199.58426	-87.79213	**193.92830**	-90.96415	224.84276	-111.42138

Supplementary Table S1. Summary of mean values of the estimation of the transition rate matrix for discrete trait evolution (Diet, Order, Habitat, Trophic Level) over 100 trees to account for phylogenetic uncertainty. Selected models based on the Akaike Information Criterion (bold). Models tested: All Rates Different (ARD), Symmetric (SYM), and Equal Rates (ER).

	Lh	Lh + Prior	No. Pram	Alpha	Sigma2
Run 1	4751.000	4751.000	4751.000	4751.000	1414.183
Run 2	4751.000	4379.304	4751.000	4751.000	2317.672

Supplementary Table S2. Effective sample size of the runs that converged from BayesTraits.

	Point est.	Upper CI
Lh	1.00	1.00
Lh+prior	1.00	1.00
No.Pram	1.00	1.00
Alpha	1.00	1.00
Sigma2	1.01	1.01

Supplementary Table S3. Gelman and Rubin's convergence diagnostic results from the two runs that displayed convergence in the trace plots. Multivariate psfr 1

ESS						
	root	deep	pelagic	reef	shelf	
var1	455.6129	3097.128	4178.163	4100.960	2743.791	
var2	216.3922	1774.421	1300.799	1521.284	1371.214	
var3	NA	1774.421	1300.799	1521.284	1371.214	
var4	NA	2834.934	5126.708	3360.384	2462.130	
	root	BP	CEPH	CR	FISH	GEN
var1	328.0183	4606.5131	1109.9138	1425.773	1221.966	1408.6730
var2	398.6594	773.9470	831.7796	513.367	1453.376	470.5297
var3	NA	773.9470	831.7796	513.367	1453.376	470.5297
var4	NA	872.6442	709.0412	2596.624	1409.481	1302.2608
	root	LP	MP	TP		
var1	617.4140	1359.0812	573.6187	539.1553		
var2	276.2039	714.5662	789.7219	1052.1904		
var3	NA	714.5662	789.7219	1052.1904		
var4	NA	2094.7203	1043.5427	866.4019		
	root	LP	MP	TP		
var1	1861.453	3730.825	3880.547	2827.750		
var2	1237.170	3466.664	2628.731	2325.155		
var3	NA	3466.664	2628.731	2325.155		
var4	NA	4938.279	5377.689	4721.908		

Supplementary Table S4. Effective sample size of the runs that converged from the ratematrix analysis. Regimes are the habitat (deep, pelagic, reef, shelf); diet (BP, CEPH, CR, FISH, GEN), and trophic level (LP, MP, TP).

Group	n	mean	median	sd	se
Carcharhiniformes	100	1.108429E-07	1.197000E-07	5.228001E-08	5.228001E-09
Hexanchiformes	100	1.136257E-06	1.014000E-06	6.470205E-07	6.470205E-08
Lamniformes	100	1.975579E-07	2.046000E-07	1.805218E-07	1.805218E-08
Orectolobiformes	100	1.439775E-06	1.115000E-06	1.342549E-06	1.342549E-07
Squaliformes	100	1.408004E-06	1.328000E-06	5.304902E-07	5.304902E-08
Squatiniformes	100	8.243343E-08	8.868000E-08	5.635927E-08	5.635927E-09
BP	100	2.686985E-07	2.236000E-07	2.966704E-07	2.966704E-08
CEPH	100	3.617352E-07	3.144000E-07	3.027426E-07	3.027426E-08
CR	100	1.621429E-07	1.067000E-07	1.834880E-07	1.834880E-08
FISH	100	1.580692E-07	1.576000E-07	1.019610E-07	1.019610E-08
GEN	100	1.366694E-07	8.851000E-08	1.467994E-07	1.467994E-08
LP	100	3.436747E-07	2.769000E-07	3.084158E-07	3.084158E-08
MP	100	1.801860E-07	1.447000E-07	1.644276E-07	1.644276E-08
TP	100	2.254861E-07	1.650000E-07	2.094166E-07	2.094166E-08
Deep	100	9.400033E-07	8.316000E-07	4.606434E-07	4.606434E-08
Pelagic	100	1.789845E-07	1.780000E-07	1.315134E-07	1.315134E-08
Reef	100	6.624241E-07	6.277000E-07	4.109013E-07	4.109013E-08
Shelf	100	1.222006E-07	1.216000E-07	7.389587E-08	7.389587E-09

Supplementary Table S5. Mean evolutionary rates obtained from the estimation on 100 random trees extracted from the posterior distribution. Each group value is indicated along with the median, standard deviation and standard error.

	Hexa	Squa	Squt	Orec	Lamn	Carc
Obs.	0.017	0.039	0.005	0.017	0.012	0.011
Bs.median	0.014	0.036	0.004	0.016	0.010	0.011
Hexa	-					
Squa	**9.3879e-32**	-				
Squt	**2.3717e-22**	**2.4493e-33**	-			
Orec	**4.3284e-10**	**2.8638e-29**	**2.4493e-33**	-		
Lamn	8.5880e-02	**1.3912e-32**	**7.2428e-33**	**1.0557e-23**	-	
Carc	**4.6727e-04**	**3.8432e-33**	**2.4493e-33**	**5.9463e-32**	6.2173e-01	-

Supplementary Table S6. Procrustes variance estimated for each order (with 3 or more species) with observed disparity and bootstrapped median (100 bootstrap replicates). Lower triangle indicates the pairwise comparison with p value after Wilcoxon test with Bonferroni correction. Hexa = Hexanchiformes; Squa = Squaliformes; Squt = Squatiniformes; Orec = Orectolobiformes; Lamn = Lamniformes; Carc = Carcharhiniformes.

	BP	CEPH	CR	FISH	GEN
Obs.	0.008	0.040	0.026	0.019	0.024
Bs.median	0.007	0.038	0.025	0.018	0.021
BP (n = 6)	-				
CEPH (n = 18)	**2.558394e-33**	-			
CR (n = 12)	**2.558394e-33**	**4.814603e-20**	-		
FISH (n = 27)	**2.558394e-33**	**1.200821e-30**	**5.383023e-14**	-	
GEN (n = 7)	**2.557385e-33**	**1.492436e-27**	**2.292704e-06**	**4.023504e-04**	-

Supplementary Table S7. Procrustes variance estimated diet groups (with more than 5 species) with observed disparity and bootstrapped median (100 bootstrap replicates). Lower triangle indicates the pairwise comparison with p value after Wilcoxon test with Bonferroni correction. BP = Big predator; CEPH = Cephalopod consumer; CR = Crustacean consumer; FISH =Fish consumer; GEN = Generalist

	TP	MP	LP
Obs.	0.019	0.030	0.044
Bs.median	0.018	0.030	0.042
TP (n = 29)	-		
MP (n = 41)	**2.984074e-27**	-	
LP (n = 19)	**5.042729e-33**	**2.715689e-25**	-

Supplementary Table S8. Procrustes variance estimated for each trophic level group with observed disparity and bootstrapped median (100 bootstrap replicates). Lower triangle indicates the pairwise comparison with p value after Wilcoxon test with Bonferroni correction. TP = Top predator; MP = Mesopredator; LP = Low level predator.

	Deep	Pelagic	Shelf	Reef
Obs.	0.042	0.010	0.015	0.026
Bs.median	0.039	0.010	0.015	0.025
Deep (n = 21)	-			
Pelagic (n = 17)	**1.537286e-33**	-		
Reef (n = 23)	**3.452424e-33**	**3.792197e-30**	-	
Shelf (n = 28)	**3.323061e-23**	**1.537286e-33**	**1.537286e-33**	-

Supplementary Table S9. Procrustes variance estimated for habitat groups with observed disparity and bootstrapped median (100 bootstrap replicates). Lower triangle indicates the pairwise comparison with p value after Wilcoxon test with Bonferroni correction.

	Df	SS	MS	Rsq	F	Z	P
log(CS)	1	0.19346	0.193457	0.07456	7.3694	3.6326	**0.001**
TR	2	0.12234	0.061168	0.04715	2.3301	2.2902	**0.01**
log(CS):TR	2	0.09994	0.049969	0.03852	1.9035	1.7833	**0.04**
Residuals	83	2.17885	0.026251	0.83977			
Total	88	2.59458					
log(CS)	1	0.19346	0.193457	0.07456	8.7516	3.9352	**0.001**
Habitat	3	0.47001	0.156671	0.18115	7.0875	6.4695	**0.001**
log(CS):Habitat	3	0.14058	0.046859	0.05418	2.1198	2.3572	**0.007**
Residuals	81	1.79053	0.022105	0.6901			
Total	88	2.59458					
log(CS)	1	0.19346	0.193457	0.07456	12.0913	4.4623	**0.001**
Order	8	1.07777	0.134722	0.41539	8.4203	6.0507	**0.001**
log(CS):Order	6	0.15538	0.025896	0.05988	1.6185	1.4889	0.079
Residuals	73	1.16798	0.016	0.45016			
Total	88	2.59458					
log(CS)	1	0.22038	0.220382	0.09879	9.3212	4.05	**0.001**
Diet	7	0.41218	0.058883	0.18478	2.4905	3.0537	**0.001**
log(CS):Diet	6	0.17956	0.029926	0.08049	1.2657	0.863	0.197
Residuals	60	1.4186	0.023643	0.63594			
Total	74	2.23071					

Supplementary Table S10. Procrustes MANCOVA results for each category covarying with the logarithm of centroid size. Bold indicates significant at $p < 0.05$. Df = Degrees of freedom; SS = Sum of squares; MS = Mean squares; Rsq = r squared; F = F value; Z = Z-score; log(CS) = logarithm of centroid size; TR = Trophic Level.

	GIC	Log-likelihood
BM	-743980.4	375819.8
OU	-756867.1	382263
EB	**-770590.6**	389125.7
lambda	-626336	316999

Supplementary Table S11. Comparison of evolutionary models by penalized likelihood with generalized information criterion (GIG). Bold indicates the best supported model. BM = Brownian Motion; OU = Ornstein-Uhlenbeck; EB = Early Burst; lambda = Pagel's lambda transformation.

Regimes	Correlation among traits	Evolutionary rates among traits		Overlap between regimes
	PC1vsPC2	PC1	PC2	PC1vsPC2
deep x pelagic	0.02158333	7.5e-05	0.9835583	**0.0027500**
deep x reef	0.036075	0.046	0.7979	**0.03625833**
deep x shelf	0.718625	2e-04	0.9080583	0.1153833
pelagic x reef	0.7284833	0.00705	0.775325	0.9880083
pelagic x shelf	0.04594167	0.0602	0.94855	**0.03086667**
reef x shelf	0.06405833	0.2207417	0.6788667	0.2367000

Supplementary Table S12. Summary statistics of the posterior distribution overlap among the rates of evolution and correlations between traits. The regimes are the four habitat groups. Bold indicates differences between posterior parameters estimates between regimes overlap.

Regimes	Correlation among traits	Evolutionary rates among traits		Overlap between regimes
BP x CEPH	0.9564583	0.8189083	0.3805417	0.9800083
BP x CR	0.8950167	0.7482583	0.59685	0.8417083
BP x FISH	0.7472833	0.1574833	0.01089167	0.32859167
BP x GEN	0.9211833	0.6123833	0.3044833	0.9733083
CEPH x CR	0.929175	0.6124583	0.7431833	0.8806500
CEPH x FISH	0.7155833	0.4358667	0.2211333	0.3881667
CEPH x GEN	0.9636833	0.9822833	0.9243	0.956675
CR x FISH	0.61035	0.9571	0.8312333	0.4398917
CR x GEN	0.9737917	0.9031583	0.9978917	0.9161667
FISH x GEN	0.6309333	0.8159833	0.838975	0.508200

Supplementary Table S13. Summary statistics of the posterior distribution overlap among the rates of evolution and correlations between traits. The regimes are the main five diet groups. BP = Big predator; CEPH = Cephalopod consumer; CR = Crustacean consumer; FISH =Fish consumer; GEN = Generalist

Regimes	Correlation among traits	Evolutionary rates among traits		Overlap between regimes
LP x MP	0.4723167	0.1115083	0.2617917	0.1410667
LP x TP	0.4378333	0.118175	0.259	0.1282833
MP x TP	0.974675	0.9919167	0.992925	0.9630083

Supplementary Table S14. Summary statistics of the posterior distribution overlap among the rates of evolution and correlations between traits. The regimes are the three trophic level groups. TP = Top predator; MP = Mesopredator; LP = Low level predator.

Supplementary References

1. Watanabe, A. (2018). How many landmarks are enough to characterize shape and size variation?. *PloS one*. **13**, e0198341 (2018).
2. Schlager, S. Morpho and Rvcg–Shape Analysis in R: R-Packages for geometric morphometrics, shape analysis and surface manipulations. in *Statistical Shape and Deformation Analysis* (eds Zheng, G. et al.) 217–256 (Academic Press, 2017).
3. Cortés, E. Standardized diet compositions and trophic levels of sharks. *ICES J. Mar. Sci.* **56**, 707–717 (1999).
4. Galili, T. dendextend: an R package for visualizing, adjusting and comparing trees of hierarchical clustering. *Bioinformatics*. **31**, 3718-3720 (2015).
5. Froese, R. & Pauly, D. FishBase. World Wide Web electronic publication www.fishbase.org (2022).
6. Froese, R. *Life-History Strategies of Recent Fishes: A Meta-Analysis.* PhD thesis, Christian-Albrecht Universität (2005).
7. Compagno, L. J. Alternative life-history styles of cartilaginous fishes in time and space. Environmental Biology of Fishes, **28**, 33-75 (1990).
8. Dulvy, N. K. et al. Extinction risk and conservation of the world's sharks and rays. *eLife* **3**, e00590 (2014).
9. Adams, D. C. & Otárola-Castillo, E. geomorph: an R package for the collection and analysis of geometric morphometric shape data. *Methods Ecol. Evol.* **4**, 393-399 (2013).
10. Kassambara, A. & Kassambara, M. A. Package 'ggpubr'. R package version 0.1 6 (R Foundation for Statistical Computing, 2020).
