# Supplementary material for: Shark mandible evolution reveals patterns of trophic and habitat-mediated diversification
Source: Commun Biol. 2023 May 8;6:496. doi: 10.1038/s42003-023-04882-3 (PMC10167336; doi:10.1038/s42003-023-04882-3)
Supplement: Supplementary file 3 — Description of Additional Supplementary Files [file 42003_2023_4882_MOESM3_ESM.pdf]

Description of Additional Supplementary Files

File name: Supplementary data 1

Description: Spread sheet of specimens, with references complementing the information of the diet stomach content. Also, indication if substitute species used when information of stomach content was not available. Associated information for averaging the raw landmark coordinates by species.

File name: Supplementary data 2

Description: Spread sheet of percentages values of prey items by species derived from the values obtained from the literature cited in supplementary data 1.

File name: Supplementary data 3

Description: Associated classifiers (factors) used for the analyses for every species after averaged landmarks. These are used in conjunction with the landmark data averaged values for each species.
