# Supplementary material for: Shark mandible evolution reveals patterns of trophic and habitat-mediated diversification
Source: Commun Biol. 2023 May 8;6:496. doi: 10.1038/s42003-023-04882-3 (PMC10167336; doi:10.1038/s42003-023-04882-3)
Supplement: Supplementary file 7 — Reporting Summary [file 42003_2023_4882_MOESM7_ESM.pdf]

nature portfolio

Corresponding author(s): Faviel A. López Romero

Last updated by author(s): Apr 24, 2023

Reporting Summary

Nature Portfolio wishes to improve the reproducibility of the work that we publish. This form provides structure for consistency and transparency in reporting. For further information on Nature Portfolio policies, see our Editorial Policies and the Editorial Policy Checklist.

Statistics

For all statistical analyses, confirm that the following items are present in the figure legend, table legend, main text, or Methods section.

n/a	Confirmed	
☐	☒	The exact sample size (*n*) for each experimental group/condition, given as a discrete number and unit of measurement
☒	☐	A statement on whether measurements were taken from distinct samples or whether the same sample was measured repeatedly
☐	☒	The statistical test(s) used AND whether they are one- or two-sided<br>*Only common tests should be described solely by name; describe more complex techniques in the Methods section.*
☐	☒	A description of all covariates tested
☐	☒	A description of any assumptions or corrections, such as tests of normality and adjustment for multiple comparisons
☐	☒	A full description of the statistical parameters including central tendency (e.g. means) or other basic estimates (e.g. regression coefficient) AND variation (e.g. standard deviation) or associated estimates of uncertainty (e.g. confidence intervals)
☐	☒	For null hypothesis testing, the test statistic (e.g. *F*, *t*, *r*) with confidence intervals, effect sizes, degrees of freedom and *P* value noted<br>*Give P values as exact values whenever suitable.*
☐	☒	For Bayesian analysis, information on the choice of priors and Markov chain Monte Carlo settings
☒	☐	For hierarchical and complex designs, identification of the appropriate level for tests and full reporting of outcomes
☐	☒	Estimates of effect sizes (e.g. Cohen's *d*, Pearson's *r*), indicating how they were calculated

Our web collection on statistics for biologists contains articles on many of the points above.

Software and code

Policy information about availability of computer code

Data collection	Landmark Editor (Version 3.0) for landmark capture. http://vertlife.org/sharktree/ for phylogenetic information.
Data analysis	R software (https://www.r-project.org/), in TreeAnnotator version 1.8.2, BayesTraits v 3.0.2 (http://www.evolution.rdg.ac.uk). All relevant packages are described in the Methods section and in the Script in Code Availability section.

For manuscripts utilizing custom algorithms or software that are central to the research but not yet described in published literature, software must be made available to editors and reviewers. We strongly encourage code deposition in a community repository (e.g. GitHub). See the Nature Portfolio guidelines for submitting code & software for further information.

Data

Policy information about availability of data

All manuscripts must include a data availability statement. This statement should provide the following information, where applicable:

- Accession codes, unique identifiers, or web links for publicly available datasets
- A description of any restrictions on data availability
- For clinical datasets or third party data, please ensure that the statement adheres to our policy

All data sources used in the study is indicated in the methods section. The ply files for the shark species can be found at https://doi.org/10.6084/m9.figshare.c.3662366.v1 and 10.18563/journal.m3.133 for C. milii.

Human research participants

Policy information about studies involving human research participants and Sex and Gender in Research.

Reporting on sex and gender	Not applicable
Population characteristics	Not applicable
Recruitment	Not applicable
Ethics oversight	Not applicable

Note that full information on the approval of the study protocol must also be provided in the manuscript.

Field-specific reporting

Please select the one below that is the best fit for your research. If you are not sure, read the appropriate sections before making your selection.

☒ Life sciences ☐ Behavioural & social sciences ☐ Ecological, evolutionary & environmental sciences

For a reference copy of the document with all sections, see nature.com/documents/nr-reporting-summary-flat.pdf

Life sciences study design

All studies must disclose on these points even when the disclosure is negative.

Sample size	The sample was chosen based on specimen availability in the collections and available CT-Scan Data at the moment.
Data exclusions	The quality of the surface reconstruction was considered to exclude specimens.
Replication	Sensitivity analyses regarding the outliers from the group were conducted. In the case of morphological disparity the groups were also subject to other metrics to check the consistency of the results.
Randomization	We used a random subsample of 100 trees from the posterior probability to estimate the morphological evolutionary rates and disparity through time.
Blinding	Blinding does not apply here. But different grouping was tested to check for consistency.

Reporting for specific materials, systems and methods

We require information from authors about some types of materials, experimental systems and methods used in many studies. Here, indicate whether each material, system or method listed is relevant to your study. If you are not sure if a list item applies to your research, read the appropriate section before selecting a response.

Materials & experimental systems

n/a	Involved in the study	
☒	☐	Antibodies
☒	☐	Eukaryotic cell lines
☒	☐	Palaeontology and archaeology
☐	☒	Animals and other organisms
☒	☐	Clinical data
☒	☐	Dual use research of concern

Methods

n/a	Involved in the study	
☒	☐	ChIP-seq
☒	☐	Flow cytometry
☒	☐	MRI-based neuroimaging

Animals and other research organisms

Policy information about studies involving animals; ARRIVE guidelines recommended for reporting animal research, and Sex and Gender in Research

Laboratory animals	No laboratory animals were used

Wild animals	All specimens are housed in collections as described in Brazeau, Martin; Kamminga, Pepijn; De Bruin, Paul W.; Geleijns, Jacob (2017): X-ray computed tomography library of shark anatomy and lower jaw surface models. figshare. Collection. https://doi.org/10.6084/m9.figshare.c.3662366.v1 and Dearden, R., Mansuit, R., Cuckovic, A., Herrel, A., Didier, D., Tafforeau, P., & Pradel, A. (2021). 3D models related to the publication: The morphology and evolution of chondrichthyan cranial muscles: a digital dissection of the elephantfish Callorhinchus milii and the catshark Scyliorhinus canicula. MorphoMuseuM.
Reporting on sex	Does not apply. The specimens were selected based on the availability in the museums collections and the literature.
Field-collected samples	No field-collected samples were used.
Ethics oversight	No ethical approval applies since the project involved exclusively museum specimens and non destrutive procedure to analyse their anatomy.

Note that full information on the approval of the study protocol must also be provided in the manuscript.
